# Malignant melanoma in a grey horse: case presentation and review of equine melanoma treatment options

**DOI:** 10.1186/2046-0481-66-22

**Published:** 2013-11-06

**Authors:** Lucy VA Metcalfe, Peter J O’Brien, Stratos Papakonstantinou, Stephen D Cahalan, Hester McAllister, Vivienne E Duggan

**Affiliations:** 1 Section of Veterinary Clinical Studies, School of Veterinary Medicine, Veterinary Science Centre, Belfield, Dublin 4, Ireland; 2 Section of Veterinary Sciences, School of Veterinary Medicine, Veterinary Science Centre, Belfield, Dublin 4, Ireland; 3 Department of Pathology and Pathogen Biology, The Royal Veterinary College, Hawkshead Lane, North Mymms, Hatfield, England

**Keywords:** Melanoma, Malignant, Benign, Metastases, Pigmented

## Abstract

A 15 year-old grey Thoroughbred gelding presented for investigation of chronic weight loss and recent onset of respiratory difficulty. Clinical examination confirmed tachypnoea with increased respiratory effort. Thoracic ultrasound examination detected pleural effusion. The dyspnoea was related to the large volume of pleural effusion and, following *post-mortem* examination, to the presence of a large mediastinal mass. Multiple pigmented masses, likely melanomas, were detected peri-anally. Thoracic radiography, cytological examination of the pleural fluid and a fine needle aspirate of a thoracic mass led to a presumptive diagnosis of malignant melanoma and this was confirmed at *post mortem* examination. Further metastatic spread to the central nervous system and right guttural pouch was also identified. In conclusion this case manifests the potential malignant behaviour of equine melanomas, and a review of proposed therapies for melanoma treatment highlights the therapeutic options and current areas of research.

## Introduction

Melanin is a pigment produced by melanocytes in the basal layer of the epidermis. It is proposed that, in grey horses, a disturbance in melanin metabolism and transfer associated with progressive greying of the hair, due to increasing age, results in intra-cellular accumulation of pigment. Excess pigment deposition then stimulates formation of new melanoblasts or increased melanoblast activity resulting in focal areas of overproduction and neoplastic transformation of melanocytes [1]. Melanocytic neoplasms have been reported to represent up to 18.7% of all equine cutaneous neoplasms [2].

Although it has been stated that 66% of equine melanomas may become malignant [3], results of more recent clinicopathological studies have varied. MacGillivray [4] reported that 14% of dermal melanomas may become malignant, but stated that this figure was likely an overestimate as benign melanomas are infrequently submitted. A later study investigating a group of 296 Lipizzaners reported a 50% incidence of melanomas and no clinical evidence of malignancy [5].

The following report describes a case of malignant melanoma with the discussion focussing on available treatment modalities and therapeutic and preventative measures currently being developed.

### Case history and clinical findings

A 15 year-old grey Thoroughbred gelding was referred to the University College Dublin Veterinary Hospital (UCDVH) for investigation of respiratory difficulty of three weeks duration. The horse had a history of insidious weight loss over the past year and had recently developed depression, inappetance, pyrexia and pectoral oedema. Pigmented peri-anal masses, likely melanomas, had been noted since purchase three years ago. Recent treatment by the referring veterinarian included a combination of antibiotics, non-steroidal anti-inflammatories and bronchodilators.

On presentation, the horse was quiet, alert and responsive with a body condition score of 3.5/9. Clinical examination revealed tachypnoea (30 breaths/min) and increased respiratory effort. Mucous membranes appeared pink, slightly tacky and capillary refill time was 3 seconds. Multiple pigmented masses, likely melanomas (approximately 1.5 cm diameter) were detected peri-anally. A small, firm, well circumscribed mass was palpable in the right parotid region. Upon auscultation, there were bilaterally increased lung sounds in the dorsal lung fields and reduced vesicular sounds bilaterally in the ventral lung fields. As the horse was exhibiting respiratory difficulty a rebreathing examination was not performed. Based on the clinical evidence of hypovolaemia, an intravenous catheter was placed and treatment begun with intravenous fluids (Compound sodium lactate, 2.5 ml/kg/hour IV). A maintenance rate was administered until further diagnostic evaluation had been performed.

#### Initial investigation

Arterial blood gas analysis parameters were within reference ranges. Haematology revealed a mild neutrophilic inflammatory response (white blood cell count 14.4 × 10^9^/L, reference range 4.4 – 9.0 × 10^9^/L; neutrophil count 12.8 × 10^9^/L, reference range 2.2-5.8 × 10^9^/L). Serum biochemistry revealed total hyperbilirubinaemia (194.2 umol/L, reference range 5-51 umol/L). Thoracic ultrasonography revealed a large volume of hypoechoic fluid present in the pleural cavity to the level of the tuber ischii bilaterally.

**Figure 1:**
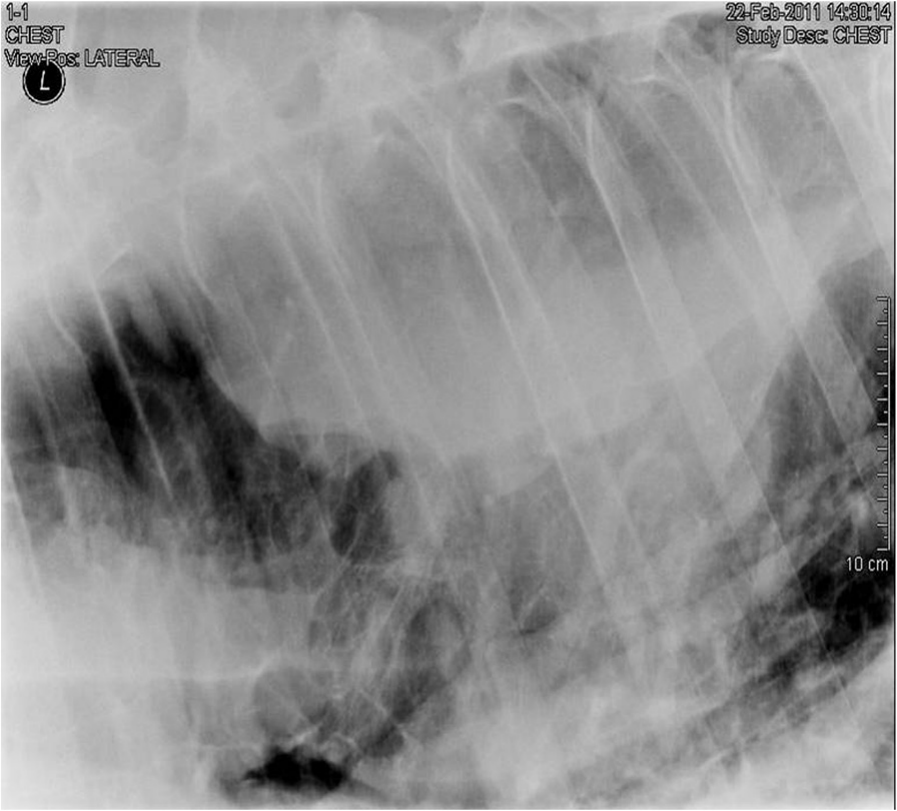
**Lateral radiograph of the left mid-dorsal thorax.** A well-marginated soft tissue opacity is visible ventral to and superimposed on the thoracic vertebrae, extending from intercostal spaces 6-13.

The left side of the chest was drained, under ultrasound guidance, with a 24 Fr thoracic trochar at the level of the elbow in the 8^th^ intercostal space (ICS); 25 litres of proteinaceous serosanguinous fluid was slowly drained. Repeat thoracic ultrasonography revealed a soft tissue structure extending from ICS 6-13 on the left side of the thorax. The structure extended ventrally from the dorsal extent of the thoracic cavity. Radiographic examination of the lung fields on the left side of the horse confirmed the location of the soft tissue opacity. The structure was superimposed over the mid-thoracic vertebral bodies and was therefore estimated to be 12 cm in dorsoventral distance (Figure 1). Fine needle aspiration of the mass was performed and the darkly pigmented tissue obtained submitted for cytological examination along with a sample of the pleural fluid.

**Figure 2:**
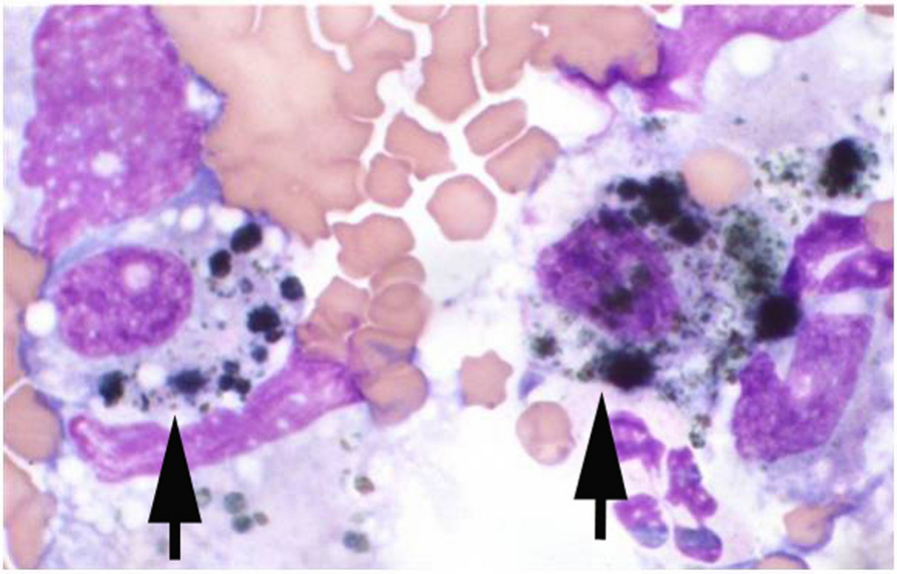
Cytospin preparation of pleural fluid showing pigmented vacuolated cells, likely melanophages (arrows).

**Figure 3:**
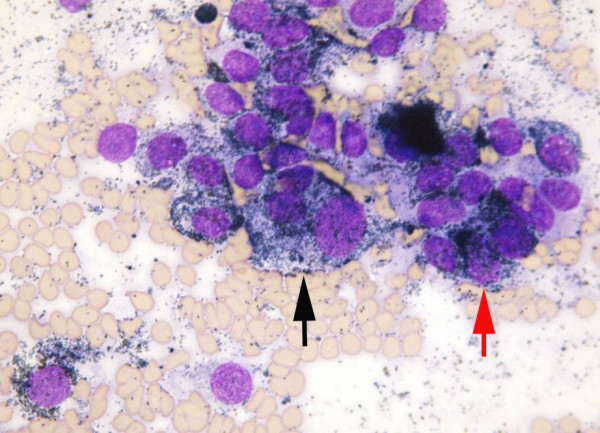
**Fine needle aspirate smear.** There are multiple cytological criteria of malignancy: anisokaryosis (black arrow), nuclear moulding (red arrow).

Cytological analysis reported the pleural fluid to be a modified transudate; large cells with abundant foamy cytoplasm containing material consistent with pigment (likely melanophages) were seen on cytospin examination (Figure 2). The fine needle aspirate smears revealed melanocytes with multiple cytological criteria of malignancy including variable pigmentation, multinucleation, moderate-to-marked anisocytosis and anisokaryosis, variable nucleus:cytoplasmic ratio and nuclear moulding (Figure 3). A presumptive diagnosis of malignant melanoma was made and following consultation with the owner a decision was made for euthanasia of the horse.

At post mortem examination large multifocal to coalescing black firm nodular masses were observed expanding and distending the mediastinum and extending multifocally throughout the pleural lining of the intercostal spaces and diaphragmatic surface. Similar masses were present in the right guttural pouch adjacent to the internal carotid artery and within the right submandibular salivary gland. A solitary black nodule was noted on the dural surface of the spinal cord in the region of T13 and within the meninges between the rostral border of the cerebellum and the dorsal aspect of the midbrain. Histological sections of perianal skin, lung tissue, pleura, brain, spinal cord and oesophagus revealed multinodular, unencapsulated, infiltrative, densely cellular, expansile neoplasms composed of spindle to epithelioid cells arranged in nests and sheets. Neoplastic cells exhibited marked pleomorphism and contained round to oval nuclei, single prominent basophilic nucleoli and eosinophilic cytoplasm containing variable amounts of melanin. Mitotic figures ranged from 2 – 5 per high powered field. Numerous tumour cell emboli were noted in lymphatics within the interlobular septae of the lung. This confirmed the diagnosis of malignant melanoma.

## Discussion

The horse described in this report had multiple small perianal masses consistent with the benign melanomas reported in approximately 80% of aged grey horses [6]. Until clinical symptoms developed and subsequently progressed there was no indication of the widespread metastatic nature of this neoplasm.

Traditionally, equine melanomas were classified based on their growth patterns into three groups: benign melanomas which grow slowly for years without metastasis, malignant transformation of a previously benign melanoma and malignant melanoma from the outset [2]. A later comprehensive study suggested that there are 4 clinically different forms: melanocytic nevi, dermal melanoma, dermal melanomatosis and anaplastic malignant melanoma [6]. Melanocytic nevi occur in young horses of all coat colours. They frequently occur singularly, in atypical sites and are benign. Dermal melanomas are generally single masses that occur in typical locations i.e. ventral tail and perineum in grey horses and surgical excision is curative [6]. Dermal melanomatosis is differentiated clinically, but is histologically indistinguishable from dermal melanoma. Melanomatosis is characterised by multiple lesions, often coalescing, in typical locations occurring in grey horses older than 15 years of age. These are not surgically curable and have a high metastatic rate [2]. Anaplastic malignant melanoma is reported in aged non-grey horses, and although rare is the most aggressive form, leading to widespread metastasis within one year of diagnosis [6].

According to the classification system described by Valentine the case described in the present report clinically represented dermal melanomatosis i.e. multiple peri-anal lesions in an aged horse [6]. However the detection of multifocal, infiltrative, expansile masses with emboli present within lymphatics, in association with a chronic lesion, suggested malignant transformation of a previously benign melanoma. These histological findings correlate with the traditional classification system described by Scott and Miller [2]. This discrepancy highlights a limitation of the current classification systems and the authors suggest that staging of equine melanomatous tumours would result in better treatment and prognostic advice being given to clients. Veterinary oncologists use the human TNM staging system to denote clinical or pathological stage of canine tumours. This system is based upon physical examination findings, diagnostic imaging and histopathology and could be adapted for use in equidae.

In contrast to reports indicating that cases of equine melanoma rarely have clinical signs referable to metastases, despite a high metastatic rate being detected at necropsy [7], euthanasia of this horse was based upon clinical symptoms secondary to melanoma metastases. The severe respiratory signs resulted from a combination of the mass effect of the large mediastinal melanoma and the consequences of a large volume of pleural effusion on lung function. The pleural fluid likely accumulated due to a combination of malignant exudation and the space-occupying effect of the mediastinal melanoma. However despite the lungs being the most common internal organ system affected [8], thoracic melanoma is rare and pleural effusion secondary to melanoma has only once been reported to the authors’ knowledge [4]. There was no evidence of melanocytes on pleural fluid cytology; highlighting the importance of additional testing to determine the underlying cause in cases presenting with pleural effusion.

By the time a definitive diagnosis in this case had been made, disease progression was too advanced to offer any reasonable treatment options. However the external melanomas were present for a number of years and earlier therapeutic options might have been possible.

## Review

Sharp surgical excision, CO_2_ laser excision and cryosurgery are often curative in cases of small well-demarcated dermal melanomas [6]. However in advanced cases the size and extensive invasion of local tissues may preclude complete removal. More recent therapeutic approaches have combined excision or surgical debulking with additional treatment modalities including cisplatin [9]. Cisplatin is a heavy metal compound that inhibits DNA synthesis. Intra-lesional cisplatin emulsion (powdered cisplatin, adrenaline and sesame oil) injected on 4 occasions, was reported to control tumour growth locally in 81% of cases; there was no effect on development of new tumours in other areas [9]. Implantation of cisplatin-containing biodegradable beads, considered to reduce operator risk, reportedly resulted in resolution of the tumour for at least 2 years in all 12 grey horses for which follow-up was available [10]. Cisplatin is likely only suitable for treating melanomas with a mass effect or early lesions prior to metastasis. There are no reports of success in treatment of the surrounding tissue with other therapeutic options i.e.5-fluorouracil, BCG and cryotherapy.

Cimetidine, a histamine H_2_-receptor antagonist, has been reported to have anti-neoplastic properties that may improve survival in patients with melanoma. Its mechanisms of action are multifactorial with histamine reported to be a potential growth factor for melanoma cells, breast and colon cancer cell lines [11]; involved in regulation of angiogenesis and an inhibitor of the immune response. Interestingly histamine levels have been shown to be nearly 3 times greater in humans with solid malignant tumours [12]. Cimetidine has also been shown to inhibit E-selectin expression resulting in decreased tumour cell extravasation [13]. Goetz [14] reported partial to complete remission in 3 horses following administration of oral cimetidine (2.5 mg/kg bwt TID for 4-12 months). Other investigators have not documented this clinical effect, possibly as the drug seems to be most effective in rapidly developing tumours [15].

Tumour cells express antigens recognisable as non-self by the immune system. These antigens are weakly immunogenic [16]. Current research is focusing on the development of immunotherapeutic approaches: immunization or immune stimulation [17,18]. Initial studies reported successful treatment of equine melanoma using a laboratory-prepared whole-cell autogenous vaccine [17] however this non-specific immunostimulation is strongly discouraged by other investigators [19]. Interestingly autologous subcutaneous implantation of freeze-thawed cubes of sarcoid tissue has been reported successful in small groups of horses [20]. To the authors’ knowledge this simple technique has been trialled, without adverse effects, in a number of practices, as adjunctive treatment of melanoma, and is an area warranting further controlled studies.

As the mechanisms of secondary immunosuppression are investigated it should become possible to target specific immunological functions. Heinzerling et al. [21] injected equine melanoma metastases with a plasmid, encoding the immunostimulatory human interleukin-12, inducing approximately 60% tumour reduction. More recently a placebo-controlled study using equine IL-12 and IL-18 reported activation of the immune system and significant tumour regression [22]. Other investigators [23] have developed an experimental protocol involving cytokine-enhanced tumour vaccination plus suicide gene therapy. This led to a significant reduction in size (50-100%) and perceived improvement in quality of life.

In the United States there is a DNA plasmid vaccine, encoding human tyrosinase (HuTyr), licensed for the treatment of canine melanoma. This xenogenic vaccine utilises the 92% homology of human and canine tyrosinase to produce a tyrosinase-specific anti-tumour response. The equine tyrosinase sequence has a 90% homology to the human sequence therefore cross-reactivity of the HuTyr DNA vaccine is an area warranting further research [24].

While the heritability of melanoma (0.36) implies a strong genetic basis for prevalence, the mode of inheritance is inconclusive [5]. Rieder et al. [25] investigated the genetic mechanisms involved in retarding metastatic events in affected grey horses. They hypothesised an evolutionary advantage not present in solid-coloured horses, which show accelerated tumour proliferation, and suggested linkage of the ‘grey-gene’ and the ‘melanoma susceptibility-gene’. This could direct selective breeding; reported to be successful in reducing melanoma incidence in pigs [26].

## Conclusions

In conclusion, veterinarians should recognise that dermal melanomas can indeed have life-threatening consequences. As a profession we are responsible for improving client awareness of the risk of malignant transformation of these ‘benign’ cutaneous tumours.

## Competing interests

The authors declare that they have no competing interests

## Authors’ contributions

LM drafted the manuscript, VD supervised clinical work-up, HM carried out the diagnostic imaging, SP and PO’B carried out the clinical pathology and SC carried out the post mortem examination. All authors read and approved the final manuscript.
